# Application of mNGS in the Etiological Analysis of Lower Respiratory Tract Infections and the Prediction of Drug Resistance

**DOI:** 10.1128/spectrum.02502-21

**Published:** 2022-02-16

**Authors:** Haibing Liu, Yue Zhang, Jun Yang, Yanfang Liu, Jianguo Chen

**Affiliations:** a Department of Clinical Laboratory, The Affiliated People’s Hospital of Jiangsu University, Zhenjiang, Jiangsu, China; b Department of Central Laboratory, The Affiliated People’s Hospital of Jiangsu University, Zhenjiang, Jiangsu, China; Montefiore Medical Center and Albert Einstein College of Medicine

**Keywords:** lower respiratory tract infections, metagenomic next-generation sequencing, receiver operating characteristic curve, lg(RPKM), genomic coverage, relative abundance

## Abstract

Lower respiratory tract infections (LRTIs) have high morbidity and mortality rates. However, traditional etiological detection methods have not been able to meet the needs for the clinical diagnosis and prognosis of LRTIs. The rapid development of metagenomic next-generation sequencing (mNGS) provides new insights for the diagnosis and treatment of LRTIs; however, little is known about how to interpret the application of mNGS results in LRTIs. In this study, lower respiratory tract specimens from 46 patients with suspected LRTIs were tested simultaneously using conventional microbiological detection methods and mNGS. Receiver operating characteristic (ROC) curves were used to evaluate the performance of the logarithm of reads per kilobase per million mapped reads [lg(RPKM)], genomic coverage, and relative abundance of the organism in predicting the true-positive pathogenic bacteria. True-positive viruses were identified according to the lg(RPKM) threshold of bacteria. We also evaluated the ability to predict drug resistance genes using mNGS. Compared to that using conventional detection methods, the false-positive detection rate of pathogenic bacteria was significantly higher using mNGS. It was concluded from the ROC curves that the lg(RPKM) and genomic coverage contributed to the identification of pathogenic bacteria, with the performance of lg(RPKM) being the best (area under the curve [AUC] = 0.99). The corresponding lg(RPKM) threshold for identifying the pathogenic bacteria was −1.35. Thirty-five strains of true-positive virus were identified based on the lg(RPKM) threshold of bacteria, with the detection of human gammaherpesvirus 4 being the highest and prone to coinfection with Pseudomonas aeruginosa, Acinetobacter baumannii, and Stenotrophomonas maltophilia. Antimicrobial susceptibility tests (AST) revealed the resistance of bacteria containing drug resistance genes (detected by mNGS). However, the drug resistance genes of some multidrug-resistant bacteria were not detected. As an emerging technology, mNGS has shown many advantages for the unbiased etiological detection and the prediction of antibiotic resistance. However, a correct understanding of mNGS results is a prerequisite for its clinical application, especially for LRTIs.

**IMPORTANCE** LRTIs are caused by hundreds of pathogens, and they have become a great threat to human health due to the limitations of traditional etiological detection methods. As an unbiased approach to detect pathogens, mNGS overcomes such etiological diagnostic challenges. However, there is no unified standard on how to use mNGS indicators (the sequencing reads, genomic coverage, and relative abundance of each organism) to distinguish between pathogens and colonizing microorganisms or contaminant microorganisms. Here, we selected the mNGS indicator with the best identification performance and established a cutoff value for the identification of pathogens in LRTIs using ROC curves. In addition, we also evaluated the accuracy of antibiotic resistance prediction using mNGS.

## INTRODUCTION

Lower respiratory tract infections (LRTIs) are prevalent worldwide, with high morbidity and mortality, especially in children, elderly, and immunocompromised populations (1, 2). The timely and accurate determination of infectious pathogens in LRTIs is difficult, as LRTIs are caused by hundreds of pathogens, including bacteria, virus, and fungi. In immunocompromised patients, almost all bacteria or fungi can be considered potential pathogens in pulmonary infections (3).

At present, conventional pathogen testing includes microbial cultures, microscopic smears, histopathology, polymerase chain reactions (PCR), nucleic acid hybridization, and serological antibody testing. However, because of the limitations of microbial cultures and microscopic smears in terms of their detection rates, speeds, and available assay targets, it is difficult to satisfy the needs of clinicians (4).

Histopathological analysis is the diagnostic gold standard for invasive fungal infections, but it is time-consuming and lacks pathogen specificity (5). PCR assays require the design of specific primers or probes for microbial pathogens, and thus, the ability to detect pathogens is limited (6). Additionally, it is difficult to accurately predict the window period for serum antibody detection (7).

Due to the lack of microbiological diagnostic methods, clinicians often prescribe antibiotics empirically to patients with negative etiological test results, which can exacerbate a reinfection and drive the emergence of antibiotic resistance and multidrug-resistant pathogens (8). Due to the above reasons, it is necessary to identify a fast and accurate method to detect multiple pathogenic microorganisms from LRTIs.

Advancements in genome sequencing technologies and bioinformatics approaches, such as metagenomic next-generation sequencing (mNGS) (9), provide powerful options for overcoming such clinical diagnostic challenges. The mNGS (also termed high-throughput sequencing technology) has high efficiency and short turnaround time and is an unbiased approach to detect all pathogens in a clinical sample. In addition to the above advantages, mNGS can provide genetic information for evolutionary tracing, the prediction of antibiotic resistance, and the presence of virulence factors and can be used to infer the concentration of pathogens based on the number of reads (10). There have been several reports on the use of mNGS for the detection of pathogenic microorganisms in infections of the central nervous system, blood, respiratory tract, and focal tissue (11–13). However, there are still many challenges preventing its use in clinical applications. One of the primary challenges is that there is no unified standard for interpreting mNGS results. There are three main indicators in the mNGS report, including the sequencing reads, the genomic coverage, and the relative abundance of each organism. It has not yet been reported which indicator is the best for distinguishing between pathogens and colonizing microorganisms or contaminant microorganisms that are present in the sample, reagents, or laboratory environment. This is particularly true for infections of the respiratory tract, which is not a sterile environment (14).

In this study, we first evaluated the performance of the above three indicators to identify pathogenic bacteria in LRTIs using receiver operating characteristic (ROC) curves and established a cutoff value for the identification of pathogens. Using the cutoff value, we analyzed the true-positive viruses in LRTIs and identified which viruses were prone to coinfection with bacteria. In addition, we also analyzed the accuracy of antibiotic resistance prediction using mNGS.

## RESULTS

### Patient demographics and detection results of different methods.

The 46 patients enrolled in our study were mainly from the respiratory department, geriatrics department, and intensive care unit (ICU), and the average age was 70.67 ± 13.33 years old. Most of the patients were male (*n* = 33, 72%); female patients accounted for 28% (*n* = 13). Among the underlying diseases, hypertension (*n* = 17, 37%), type 2 diabetes (*n* = 9, 20%), and chronic obstructive pulmonary disease (COPD) (*n* = 8, 17%) were most prevalent. A total of 38 strains of bacteria, 1 strain of Aspergillus fumigatus, and 2 strains of human respiratory syncytial virus (RSV) were detected using conventional methods, but 63 strains of bacteria, 1 strain of Aspergillus fumigatus, and 61 strains of viruses were identified by mNGS (Table 1).

**TABLE 1:** Characteristics and detection results of patients^*a*^

Patient ID	Sex	Age (yr)	Underlying disease(s)	Detection results for:		
				Conventional detection	mNGS test	
P1	Male	78	Cerebral infarction, hypertension	Pce	Pce/HHV-7	
P2	Male	56	Hypertension	Pce	Pce/Aba/Kpn	
P3	Male	81	Anemia	Pae/Aba/Pma	Pae/Aba/Pma	
P4	Male	80	COPD	Pae	Pae/RhV-A	
P5	Male	73	Senile dementia	Pae/Pma	Pae/Pma/Kpn/HHV-1/EBV/CMV/HCov-NL63	
P6	Male	70	Hypertension	Spn	Spn/RSV-B	
P7	Male	62	Hypertension, type 2 diabetes	Pae/Pst/Bca	Pae/Pst/Bca/Eco/EBV	
P8	Male	60	Cerebral infarction	Pae	Pae/Sau/Kpn/Eco	
P9	Male	92	COPD/hypertension, type 2 diabetes	Eco	Eco/Kpn	
P10	Male	78	Hypertension, type 2 diabetes	Aba/Pma/Kpn	Aba/Pma/Kpn/EBV	
P11	Male	59	Hypertension	Aba/Pma	Aba/Pma/CMV	
P12	Female	90	Pulmonary heart disease	Kpn	Kpn/Sgc/HHV-1/HCoV-HKU1/EBV/HHV-7/RhV-A	
P13	Female	86	Hypertension	Aba	Aba/HHV-1/EBV/RhV-A	
P14	Female	92	Type 2 respiratory failure	Sau/Sma/Pae	Sau/Sma/Pae/Eco/Kpn/EBV	
P15	Female	55	Coronary heart disease	Aba	Aba/Pae/Sau EBV	
P16	Male	85	Liver cirrhosis	Sau/Pma	Sau/Pma	
P17	Male	65	None	Sau	Sau/Spy/hMPV	
P18	Male	94	COPD	Aba/Pma/Pae	Aba/Pma/Pae/Eco/Kpn/Sma/EBV	
P19	Male	47	Hypertension	Spn/Eco/RSV	Spn/Eco/CMV/Nab/RSV-B/RhV-B/HRV3	
P20	Male	53	None	Acid-fast bacillus	Mtu/Sau/HHV-7	
P21	Female	63	Type 2 diabetes	None	HHV-1/EBV/HHV-7/Cpt
P22	Male	55	Pleural effusion	RSV	RSV-B/hMPV
P23	Male	77	Type 2 diabetes, hypertension	None	EBV
P24	Male	36	Ankylosing spondylitis	Aspergillus fumigatus	Sau/HHV-7/hMPV/HCov-NL63/Aspergillus fumigatus
P25	Female	65	Hypertension	None	Cpt
P26	Female	70	Type 2 diabetes	Brevundimonas diminuta	None
P27	Male	62	None	Kpn	CMV
P28	Female	73	Hypertension	None	None
P29	Male	62	COPD	None	EBV/CMV
P30	Male	62	Hypertension	None	Hin/HHV-7/EBV
P31	Male	83	Hypertension, COPD	None	Kpn/EBV/HHV-7
P32	Female	57	SLE	None	Sau/HHV-1/EBV
P33	Female	70	None	Eco	HPV-B19
P34	Male	75	Type 2 diabetes	None	None
P35	Male	76	Anemia	None	HHV-1/EBV
P36	Male	85	Type 2 respiratory failure	None	Hin/Bca
P37	Male	73	Hypertension	None	EBV/CMV/HHV-7
P38	Male	51	Type 2 diabetes	None	EBV/HHV-7
P39	Female	84	COPD, hypertension	None	Spn/Hin
P40	Male	86	COPD	Rhizobium radiobacter	EBV
P41	Male	90	None	None	EBV/HHV-7 HCov-NL63
P42	Male	60	Pulmonary heart disease	None	HHV-7
P43	Male	71	Type 2 diabetes	None	HHV-1/HHV-7
P44	Female	67	COPD/Hypertension	None	RhV-A
P45	Female	68	None	None	None
P46	Male	74	Coronary heart disease	None	None

a COPD, chronic obstructive pulmonary disease; Aba, Acinetobacter baumannii; Eco, Escherichia coli; Pce, Burkholderia cenocepacia; Kpn, Klebsiella pneumoniae; Pma, Stenotrophomonas maltophilia; Spn, Streptococcus pneumoniae; Pae, Pseudomonas aeruginosa; Pst, Providencia stuartii; Sau, Staphylococcus aureus; Bca, Moraxella catarrhalis; Sma, Serratia marcescens; HHV-7, human betaherpesvirus 7; RhV-A, rhinovirus A; RhV-B, rhinovirus B; HHV-1, human alphaherpesvirus 1; EBV, human gammaherpesvirus 4; CMV, human betaherpesvirus 5; HCov-NL63, human coronavirus NL63; RSV-B, human respiratory syncytial virus B; Sgc, Streptococcus agalactiae; HCoV-HKU1, human coronavirus HKU1; Spy, Streptococcus pyogenes; hMPV, human metapneumovirus; Nab, Nocardia abscessus; Mtu, Mycobacterium tuberculosis; SLE, systemic lupus erythematosus; Cpt, Chlamydia psittaci; Hin, Haemophilus influenzae; HPV-B19, human parvovirus B19.

### Identification of bacteria by conventional microbiological detection methods and mNGS.

**FIG 1:**
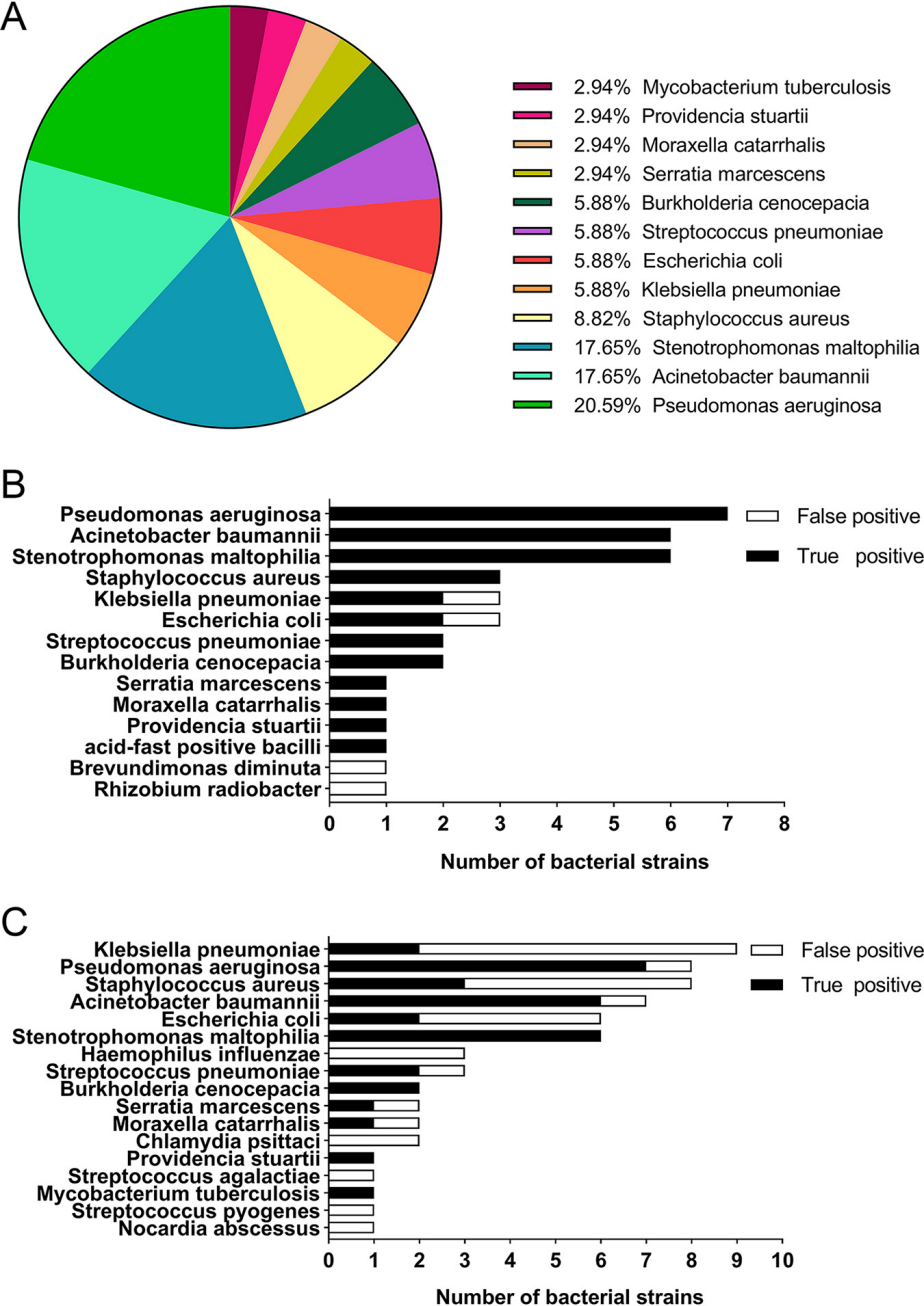
Identification of pathogenic bacteria by conventional methods and mNGS. (A) The pathogen distribution of 34 true-positive pathogenic bacteria. (B) Histogram of conventional methods to detect pathogenic bacteria. (C) Histogram of mNGS detection for pathogenic bacteria.

According to the comprehensive evaluation, 20 patients were diagnosed with bacterial infections of the lower respiratory tract, and others were diagnosed with nonbacterial infections. There was no statistical significance in demographic characteristics between the two groups (Table 2). Of the lower respiratory tract samples from these patients, 37 were sputum samples and the others were bronchoalveolar lavage fluid (BALF). All specimens were detected by conventional methods, and a total of 38 bacteria were identified in 24 samples. A total of 34 strains of bacteria were the true-positive pathogenic bacteria (Fig. 1A), and 4 strains (including 1 strain each of Escherichia coli, Klebsiella pneumoniae, Brevundimonas diminuta, and Rhizobium radiobacter) were considered false-positive pathogens, accounting for 10.5% of the 38 bacteria. The majority of specimens (11 cases) contained a single pathogen, while 4 samples had two pathogens, and the remaining 5 cases had three pathogenic bacteria. An acid-fast bacillus was detected using acid-fast staining. The most frequently detected bacteria were Pseudomonas aeruginosa (seven cases), followed by Acinetobacter baumannii (six cases) and Stenotrophomonas maltophilia (six cases) (Fig. 1B).

**TABLE 2:** Demographic characteristics of bacterial and nonbacterial infections of the lower respiratory tract^*a*^

Group	Age (yr)	Sex		Department			Underlying disease(s) presence	
		Male	Female	GER	RES	ICU	Yes	No
Bacterial infections	72.80 ± 14.82	16 (80%)	4 (20%)	4 (20%)	11 (55%)	5 (25%)	18 (90%)	2 (10%)
Nonbacterial infections	69.04 ± 12.09	17 (65.38%)	9 (34.62%)	4 (15%)	19 (73%)	3 (12%)	22 (85%)	4 (15%)
*P* value	0.348	0.336		0.391			0.684	

a GER, geriatrics department; RES, respiratory department; ICU, intensive care unit.

The lower respiratory tract specimens of the 46 patients were also sequenced by mNGS. A total of 63 bacteria were identified from 28 specimens, while 29 strains (46%) were false-positive pathogenic bacteria. The most commonly detected bacteria were K. pneumoniae (nine cases), followed by Staphylococcus aureus (eight cases), Pseudomonas aeruginosa (eight cases), and Acinetobacter baumannii (seven cases) (Fig. 1C).

### Evaluating the performance of the sequencing reads, the genomic coverage, and the relative abundance of each organism for the identification of pathogens.

**FIG 2:**
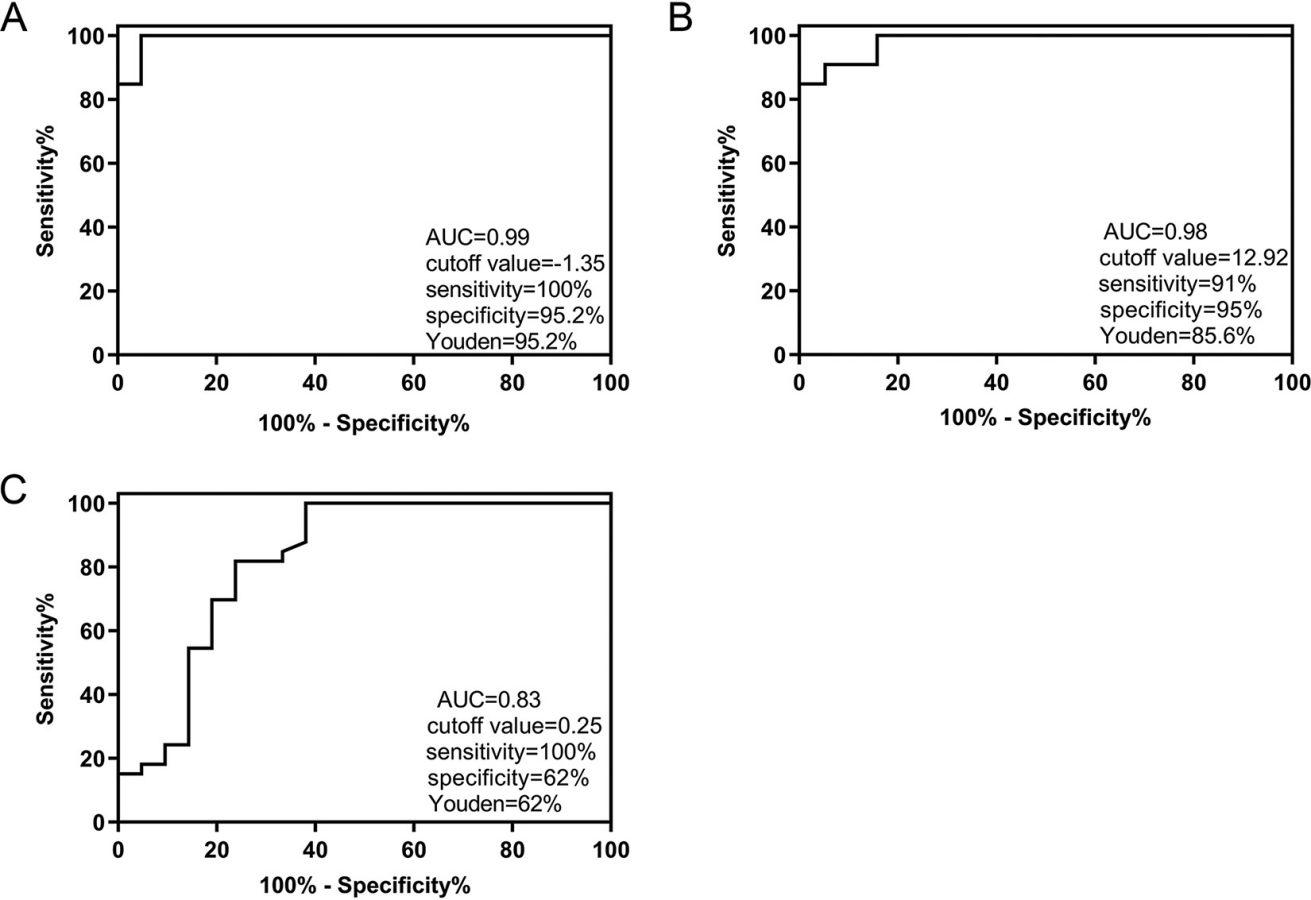
Evaluating the performance of (A) lg(RPKM), (B) genomic coverage, and (C) relative abundance for distinguishing between the true- and false-positive pathogenic bacteria groups using ROC curves.

**FIG 3:**
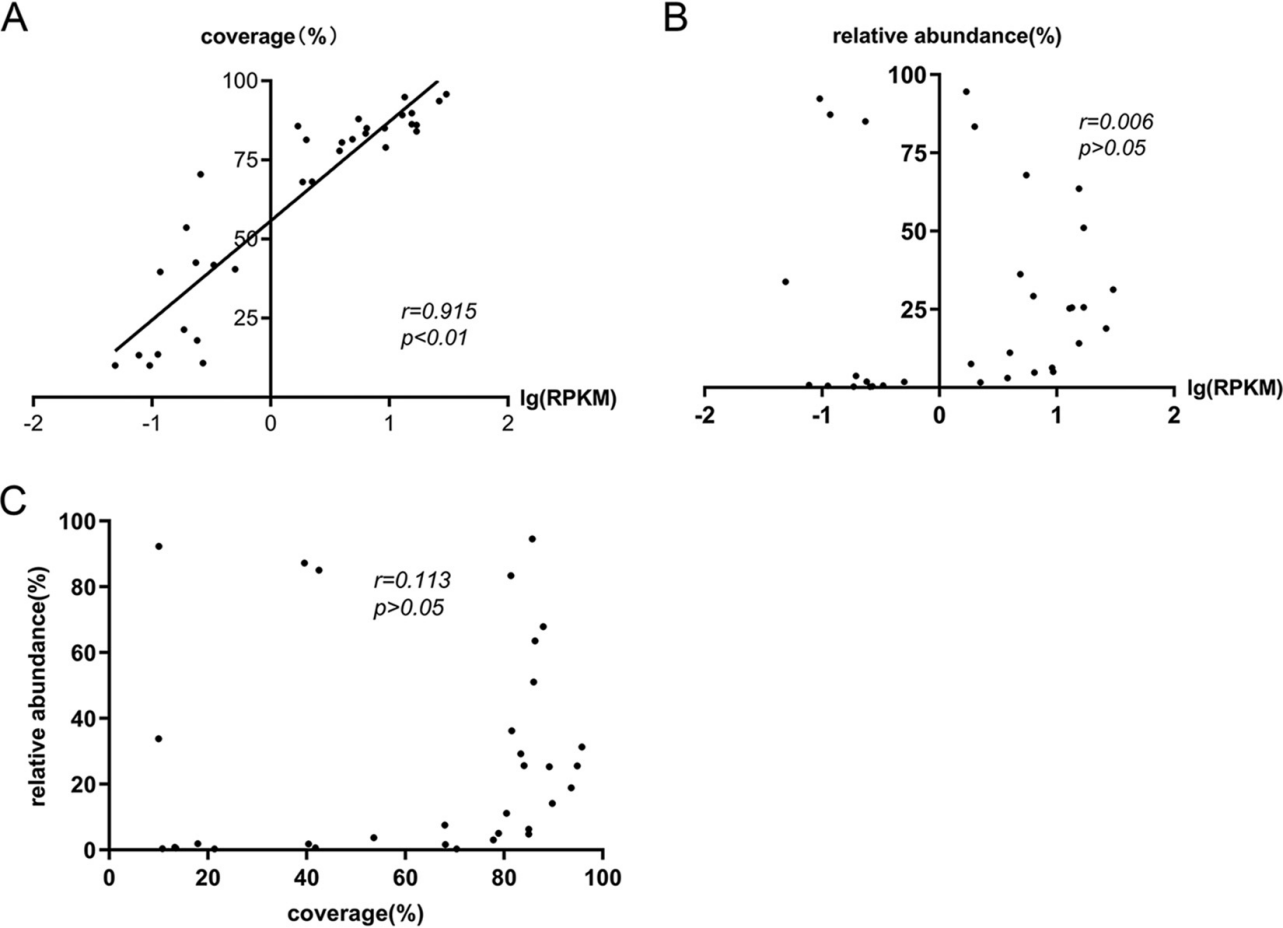
The correlation among the three indicators was analyzed using Spearman’s method. (A) A significant positive correlation was observed between lg(RPKM) and genomic coverage. (B) No significant correlation was observed between the relative abundance and lg(RPKM). (C) No significant correlation was observed between the relative abundance and genomic coverage.

With the final clinical diagnosis as the gold standard, ROC curves were used to evaluate the performance of the sequencing reads, the genomic coverage, and the relative abundance of each organism in predicting true-positive pathogenic bacteria. To rule out the effect of sequencing depth and gene length in different pathogens, the number of sequencing reads was replaced with the logarithm of reads per kilobase per million mapped reads [lg(RPKM)], and the calculation for RPKM was gene reads/[the total mapped reads (millions) × gene length (KB)]. As shown in Table 3, the lg(RPKM), genomic coverage, and relative abundance of the true-positive pathogenic bacteria group were significantly higher than those of the false-positive pathogenic bacteria group (*P* < 0.01). From the ROC curve of the three indicators, we found that the area under the curve (AUC) of the lg(RPKM) was the largest (0.99), followed by that for genomic coverage (0.98) and the relative abundance (0.83). The corresponding cutoff values were −1.35, 12.92, and 0.25, respectively (Fig. 2). The correlation between the three indicators of true-positive pathogenic bacteria was also analyzed using Spearman’s method, and a significant positive correlation was observed between the lg(RPKM) and genomic coverage (*r* = 0.951). There was no significant correlation between relative abundance and the other two indicators (Fig. 3).

**TABLE 3:** Comparison of three indicators between true- and false-positive pathogenic bacteria (median [25th percentile, 75th percentile])

Group	lg(RPKM)	Coverage (%)	Relative abundance (%)
True positive	0.35 (−0.63, 1.04)	78.92 (40.02, 85.87)	14.10 (1.85, 43.60)
False positive	−2.15 (−2.55, −1.55)	2.94 (0.60, 7.02)	0.20 (0.10, 2.20)
*P* value	0.00	0.00	0.00

### Analysis of the true-positive viruses based on mNGS results.

**FIG 4:**
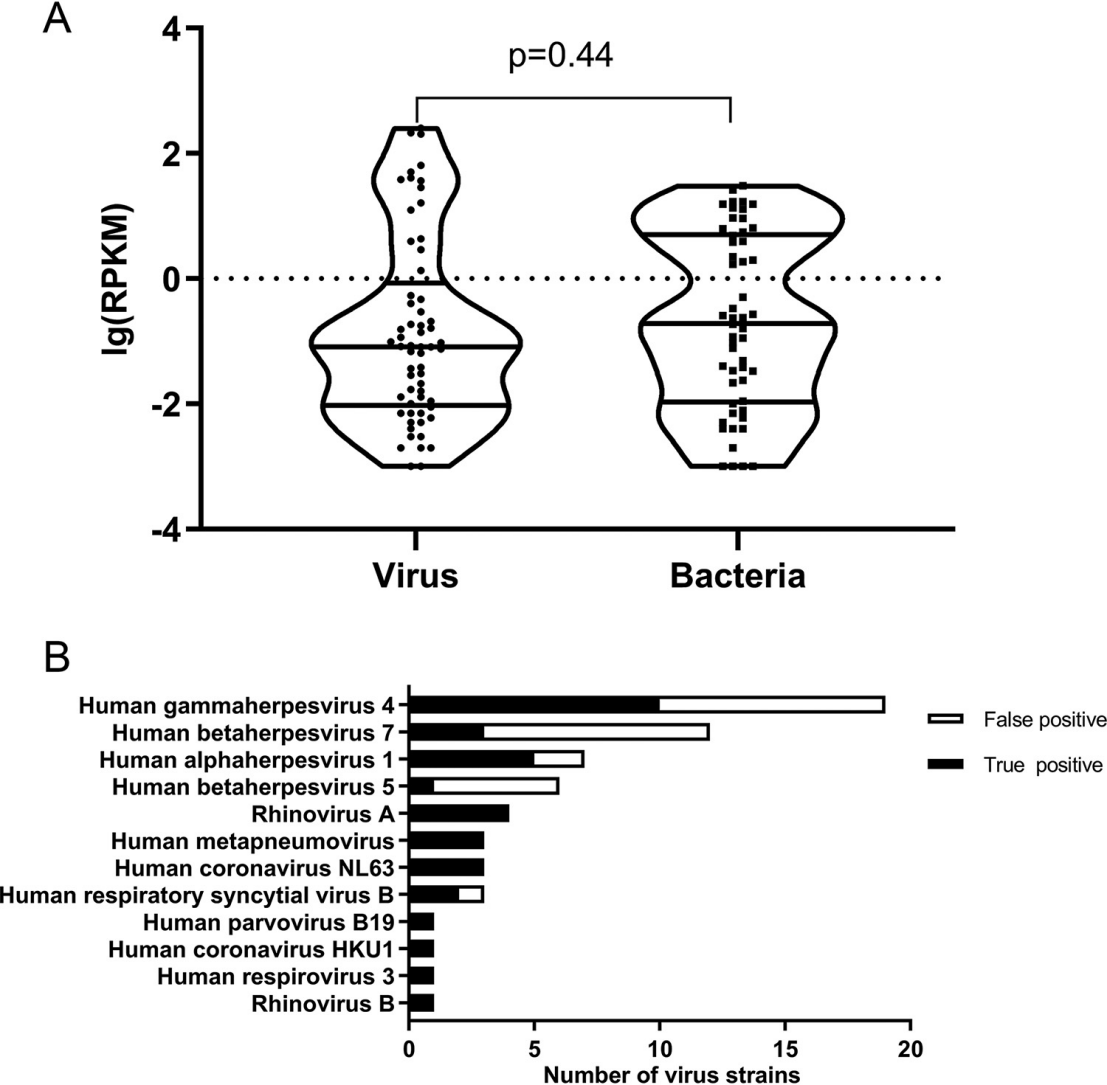
Analysis of the true-positive viruses based on mNGS results. (A) There was no statistically significant difference between the lg(RPKM) of bacteria and viruses. (B) Identification of the true-positive viruses based on the bacterial lg(RPKM) threshold.

**FIG 5:**
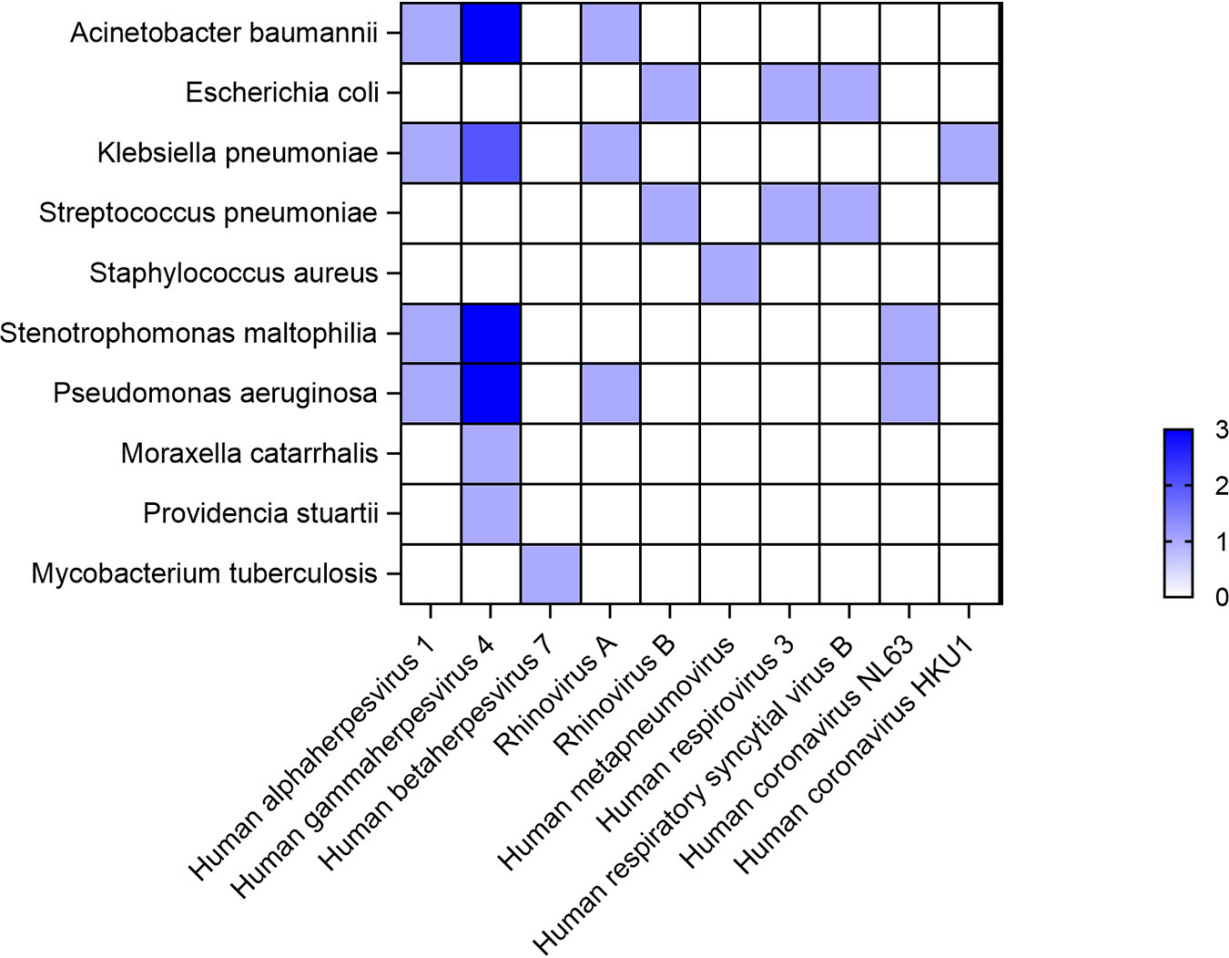
Heat map showing the trends in coinfections between bacteria and viruses.

Because of the best identification performance, the lg(RPKM) was used to identify true-positive viruses. As shown in the Fig. 4A, no statistically significant difference was observed between the lg(RPKM) of bacteria and viruses, and thus, we used the bacterial lg(RPKM) threshold to identify the infectious viruses. A total of 61 viruses were identified by mNGS, 35 of which were considered to be true-positive viruses according to the lg(RPKM) threshold and the rest of which were considered to be false positives. The most frequent true-positive virus was human gammaherpesvirus 4 (10 cases), followed by human alphaherpesvirus 1 (5 cases). Human gammaherpesvirus 4 and human betaherpesvirus 7 accounted for 33% of the false-positive viruses (Fig. 4B). In addition, we studied which viruses were prone to coinfection with particular bacteria. As shown in Fig. 5, human gammaherpesvirus 4 was most likely to be coinfected with bacteria and 6 of 10 (60%) strains of human gammaherpesvirus 4 were coinfected with 13 strains of bacteria.

### Performance of mNGS in antimicrobial resistance prediction.

The resistance genes of bacteria were predicted based on the mNGS data. We also investigated the consistency between antibiotic resistance genes and the results of antimicrobial susceptibility tests (AST). As shown in Table 4, the drug resistance genes *bla*_OXA-23_, *bla*_OXA-51_, and *bla*_TEM_ were detected in four strains of *A. baumannii*, while only *bla*_OXA-23_ and *bla*_OXA-51_ were detected in one strain of *A. baumannii*. Enzymes encoded by *bla*_OXA-23_ and *bla*_OXA-51_ belong to carbapenem-hydrolyzing class D β-lactamases, which can inactivate penicillin, cephalosporin, and carbapenem and cannot typically be inhibited by clavulanic acid, sulbactam, or tazobactam. The results of the drug sensitivity tests showed that all five strains of *A. baumannii* were resistant to ampicillin/sulbactam (SAM), piperacillin-tazobactam (TZP), ceftazidime (CAZ), cefepime (FEP), ceftriaxone (CRO), and imipenem (IPM).

**TABLE 4:** Drug resistance genes of A. baumannii predicted by mNGS and the corresponding AST^*a*^

Patient ID	Resistance genes	Resistance to drug:					
		SAM	TZP	CAZ	CRO	FEP	IPM
P3	*bla*_OXA-23_, *bla*_OXA-51_, *bla*_TEM_	R	R	R	R	R	R
P11	*bla*_OXA-23_, *bla*_OXA-51_, *bla*_TEM_	R	R	R	R	R	R
P13	*bla*_OXA-23_, *bla*_OXA-51_	R	R	R	R	R	R
P15	*bla*_OXA-23_, *bla*_OXA-51_, *bla*_TEM_	R	R	R	R	R	R
P18	*bla*_OXA-23_, *bla*_OXA-51_, *bla*_TEM_	R	R	R	R	R	R

*^a^*R, resistant.

The class A β-lactamase resistance genes were detected in four of the six strains of *Enterobacteriaceae*, including *bla*_CTX-M_, *bla*_SHV_, *bla*_TEM_, and *bla*_KPC_. The enzymes encoded by *bla*_CTX-M_, *bla*_SHV_, and *bla*_TEM_ are extended-spectrum-lactamases (ESBLs), which can hydrolyze penicillin (ampicillin [AMP]), cephalosporin (cefazolin [CZO], CAZ, CRO), and monobactam (aztreonam [ATM]) and can be inhibited by enzyme inhibitors. KPC carbapenemases encoded by *bla*_KPC_ are resistant to penicillin, cephalosporin, monobactam, and carbapenems; however, newer β-lactamase inhibitors can inhibit them, such as avibactam, relebactam, and vaborbactam. As shown in Table 5, the presence of antibiotic resistance genes was consistent with the results of drug sensitivity tests.

**TABLE 5:** Drug resistance genes of *Enterobacteriaceae* species predicted by mNGS and the corresponding AST^*a*^

Patient ID	Bacteria	Resistance gene(s)	Resistance to drug:								
			SAM	TZP	CAZ	CRO	FEP	IPM	AMP	CZO	ATM
P9	E. coli	*bla*_CTX-M_	R	S	R	R	I	S	R	R	R
P19	E. coli	*bla*_CTX-M_, *bla*_TEM_	R	S	R	R	R	S	R	R	R
P10	K. pneumoniae	*bla*_CTX-M_, *bla*_SHV_, *bla*_TEM_, *bla*_KPC_	R	R	R	R	R	R		R	R
P14	Serratia marcescens	*bla*_CTX-M_, *bla*_SHV_, *bla*_TEM_		R	R	R	R	S			R

*^a^*R, resistant; I, intermediate; S, susceptible

The *mecA* gene encodes a low-affinity penicillin-binding protein (PBP2a) that confers resistance to the entire class of β-lactams, except for ceftaroline and ceftobiprole. *mecA* was detected in all three strains of S. aureus, and those strains were resistant to penicillin (PEN) and oxacillin (OXA) (Table 6).

**TABLE 6:** Drug resistance genes of S. aureus predicted by mNGS and the corresponding AST^*a*^

Patient ID	Resistance to drug:	
	PEN	OXA
P14	R	R
P16	R	R
P17	R	R

*^a^*All three resistance genes are *mecA*. R, resistant.

## DISCUSSION

The detection of pathogenic bacteria by traditional methods is limited due to the use of broad-spectrum antibiotics, as well as the presence of bacteria that are fastidious or slow growing. By directly sequencing the DNA or RNA from samples, mNGS effectively overcomes the deficiencies of traditional detection methods. In this study, an acid-fast bacillus was detected using acid-fast staining, but it was unclear if the bacterium was Mycobacterium tuberculosis due to its slow growth phenotype. The bacterium was subsequently identified as Mycobacterium tuberculosis using mNGS (634 reads). Mycobacterium tuberculosis was considered present if at least one read was aligned at either the species or genus level due to difficulties in DNA extraction and the possibility of contamination (15). The detection rate of false-positive pathogenic bacteria by mNGS was significantly higher than that by conventional methods. Thus, it is important to distinguish between true- and false-positive pathogenic bacteria when interpreting mNGS results.

We used lg(RPKM), genomic coverage, and relative abundance as tools to identify true-positive pathogenic bacteria from mNGS data and evaluated their identification using ROC curves. An identification cutoff was also established. From our analysis, it was concluded that the lg(RPKM) and genomic coverage could identify true-positive pathogenic bacteria, with the performance of lg(RPKM) being the best. It was also seen from the correlation analyses that no significant correlation was present between the relative abundance and lg(RPKM) of pathogenic bacteria, mainly because the relative abundance was influenced by the total number of bacterial genomes in the sample. Therefore, the relative abundance was not ideal for the identification of true-positive and false-positive pathogenic bacteria.

The traditional methods for detecting respiratory viruses are based on virus-infected cell cultures, immunology-related reactions between antigens and antibodies, or PCR analyses (16). Virus isolation and culture is considered the gold standard for the identification of respiratory viruses (17), while its 4-week time requirement is too long to be used in clinical laboratories. The disadvantages of immunology-related reactions can lead to false-negative results during the window period (18). PCR is a targeted detection method but cannot be used for the detection of unknown viruses (19). Thus, the application of mNGS overcomes such problems for the identification of respiratory viruses.

Due to the limitations of detection methods in our study (indirect immunofluorescence), most respiratory viruses were not detected by traditional detection methods. However, mNGS greatly improved the detection rate of respiratory virus and effectively made up for the limitations of traditional detection methods for unknown viruses. In our study, herpesvirus was the predominant true-positive virus, accounting for 54.3% of the total true-positive viruses. It has been reported that rhinovirus, influenza A virus, and adenovirus were the main pathogenic viruses of community-acquired pneumonia (20), and we could conclude from our research that herpesvirus was the main virus detected in hospitalized patients. Twenty-five false-positive herpesviruses were identified, accounting for 96.2% of the total false-positive viruses. False positives were caused by the different genome lengths of various viruses (genome length of herpesviruses > 150 Kb, genome length of nonherpesviruses ≤ 30 Kb). The longer the viral genome, the more reads were produced, and the higher the false-positive rate.

According to previous studies, respiratory viruses can influence the pathogens of pneumonia by altering the carrying structure of bacteria in the upper respiratory tract and the colonization of the lower respiratory tract by specific bacteria (2). Thus, we investigated which viruses were prone to coinfection with particular bacteria. Our findings showed that human gammaherpesvirus 4 was prone to coinfection with P. aeruginosa, A. baumannii, and S. maltophilia. Another study also reported that human gammaherpesvirus 4 was associated with fever or acute exacerbation of chronic lung disease (21), possibly due to the bacteria with which the patients were coinfected.

The traditional ASTs include disk diffusion and MIC methods, which are too time-consuming to be used for precision medicine. Predicting resistance genes by mNGS provides us with a new strategy to infer antimicrobial susceptibility. In our study, the presence of drug-resistant genes was consistent with the results of ASTs. Additionally, the drug resistance genes identified by mNGS were associated with bacterial susceptibility to β-lactam antibiotics, while genes that conferred resistance to other classes of antibiotics were not detected. Additionally, five strains of Pseudomonas aeruginosa that were resistant to carbapenem antibiotics did not contain genes associated with drug resistance. That finding may be due to the following reasons: (i) there is not a standard database that contains all validated antimicrobial resistance genes or point mutations in genes that are associated with antimicrobial resistance, (ii) predicting antimicrobial resistance accurately using mNGS is difficult due to the lack of knowledge of drug resistance caused by genetic mutations and the emergence of new resistance mechanisms, and (iii) increased expressions of intrinsic resistant genes (e.g., those encoding efflux pumps, outer membrane proteins, and intrinsic β-lactamase) can also lead to antimicrobial resistance (22), especially in P. aeruginosa and A. baumannii. Thus, many challenges remain in inferring antimicrobial susceptibility using mNGS data.

This study had limitations. Fungal pathogens were not analyzed due to their small sample size, most of the true-positive viruses identified by the bacterial lg(RPKM) threshold could not be confirmed by other methods, and the clinical significance of the herpesvirus detections remained to be determined. However, despite this, our study still provided a new perspective on the applicability of mNGS in LRTIs. Compared with conventional microbiological detection methods, mNGS has multiple advantages. Although many challenges remain necessary to overcome for its use in clinical applications, mNGS will be a revolutionary technology for clinical microbiological diagnostics.

## MATERIALS AND METHODS

### Ethics statement.

This project was reviewed and approved by the Ethical Review Committee of Zhenjiang First People’s Hospital. An ethics review was exempted as the study was retrospective and the patients were anonymized.

### Specimen collection and processing.

We retrospectively analyzed the clinical data of 46 patients in our hospital who were suspected to have LRTIs. The lower respiratory tract specimens, including sputum or bronchoalveolar lavage fluid (BALF), from these patients were tested simultaneously by conventional microbiological detection methods and mNGS. The sputum or BALF of each patient was divided into two portions; one was sent to the clinical microbiology laboratory for conventional microbiological detection, and the other was sent to a commercial laboratory for mNGS testing.

### Conventional microbiological detection.

In the clinical microbiology laboratory, specimens were used for special pathological staining and semiquantitative culture. Gram staining (BASO), acid-fast staining (BJ-ec), and lactic acid phenol cotton blue staining (RICH SCIENCE) were performed to identify bacteria, Mycobacterium tuberculosis complex, and fungi, respectively. Bacterial culture was performed by inoculating specimens on blood agar plates (CHROMagar), MacConkey agar (CHROMagar), and chocolate agar (CHROMagar) and incubating at 35°C for 24 to 48 h. If a typical colony of pathogenic bacteria grew, bacterial identification and drug sensitivity tests were carried out using the Vitek 2 compact system (bioMérieux). Sabouraud agar plates (CHROMagar) were used for fungi culture at 28°C for 7 days. Filamentous fungi were identified according to their morphology. Influenza A/B, parainfluenza 1/2/3, human respiratory syncytial virus (hRSV), adenovirus, Mycoplasma pneumoniae, *Chlamydia*, and Legionella pneumophila were detected by indirect immunofluorescence (Euroimmun).

### The mNGS test.

Specimens (3 mL) used for mNGS were collected in sterile containers, which were then sealed and placed in a foam box containing an ice pack. Specimens were then immediately transported to a commercial laboratory (Vision Medicals) for pathogen testing. Once the laboratory received the specimens, sample processing, nucleic acid extraction, DNA library preparation, high-throughput sequencing, bioinformatics analysis, and the interpretation of mNGS data were performed according to the laboratory’s standard operating procedures. To detect the pathogens as much as possible, DNA and RNA sequencing were performed simultaneously. The total DNA and RNA from all samples were extracted using a QIAamp UCP pathogen DNA kit (Qiagen) and QIAamp viral RNA kit (Qiagen), respectively. Human DNA was removed using Benzonase (Qiagen) and Tween 20 (Sigma), and rRNA was removed by a Ribo-Zero rRNA removal kit (Illumina). cDNA was synthesized by reverse transcription. Libraries were constructed using a Nextera XT DNA library prep kit (Illumina), and the quality of libraries was assessed by Qubit dsDNA HS assay kit and high sensitivity DNA kit (Agilent) on an Agilent 2100 Bioanalyzer. Sequencing was performed using Illumina Nextseq CN500. Peripheral blood mononuclear cell (PBMC) samples with 10^5^ cells/mL from healthy donors were used as a negative control in parallel with each batch, and the same protocol was performed alongside the specimens to serve as nontemplate controls (23). The high-quality data were obtained by removing low-quality reads, adapter contamination, duplicate reads, and reads shorter than 50 bp using Trimmomatic (24). Low-complexity reads were removed by Kcomplexity with default parameters (25). Human sequence data were identified and excluded by mapping to a human reference genome (hg38) using Burrows-Wheeler Aligner software (26). A set of criteria similar to the National Center for Biotechnology Information (NCBI) criteria (https://www.ncbi.nlm.nih.gov/assembly/help/anomnotrefseq/) were designed for selecting representative assembly for microorganisms (bacteria, viruses, fungi, protozoa, and other multicellular eukaryotic pathogens) from the NCBI nucleotide and genome databases. Pathogen lists were selected according to Johns Hopkins ABX Guide, Manual of Clinical Microbiology, and clinical case reports or research articles published in current peer-reviewed journals. The final database included about 13,000 genomes. Microbial reads were then aligned to database with SNAP v1.0 beta.18. Virus-positive was defined as the coverage of three or more nonoverlapping regions on the genome. Other pathogens were considered to be positive detection if the reads per million (RPM) ratio or RPM-r (the RPM of a given species or genus in the clinical sample divided by the RPM in negative control) was ≥5 (23). Sequencing reads, genomic coverage, the relative abundance of each organism, and drug resistance genes were included in the mNGS report.

### Diagnosis of lower respiratory tract infections.

The final clinical diagnoses of the 46 patients were confirmed based on the comprehensive evaluation of clinical symptoms, X-ray evidence, traditional microbiological tests, mNGS, serological examinations [including the fungus (1-3)-β-d-glucan test, serum cryptococcal capsular polysaccharide antigen test, and Mycoplasma pneumoniae serological antibody detection], and other clinical examinations (the galactomannan antigen detection test). The pathogens detected by traditional tests or mNGS were considered true positives only if they were consistent with the final clinical diagnosis; otherwise, they were considered false positives (colonizing microorganism or contaminant microorganism).

### Statistical analysis.

The data were analyzed using SPSS 25.0 software. The Shapiro-Wilk test was used to determine whether the quantitative data conformed to a normal distribution, and the Student’s *t* test and the Wilcoxon rank test were used to compare two groups that were in normal distribution or not in normal distribution, respectively. Pearson chi-squared (χ^2^) test or Fisher’s exact test was used for the comparison of frequencies of the categorical data. The correlation between two different indicators was analyzed and expressed as Spearman’s *r* values. An ROC curve was drawn to choose the best indicator of the true-positive specific pathogen. A *P* value of <0.05 was considered statistically significant.
